# The Thirty-Second Long Fox Memorial Lecture: The Social Aspects of Acute Rheumatism

**Published:** 1944

**Authors:** C. Bruce Perry

**Affiliations:** Professor of Medicine in the University of Bristol


### The Bristol Medico-Chirurgical Journal

*" Scire est nescire, nisi id me*
*Scire alius sciret."*

SUMMER, 1944.

### THE THIRTY-SECOND LONG FOX MEMORIAL LECTURE:

BY

Professor C. Bruce Perry, M.D., F.R.C.P.,
*Professor of Medicine in the University of Bristol.*

DELIVERED IN THE UNIVERSITY OF BRISTOL ON TUESDAY, 26th OCTOBER, 1943,

ON

### THE SOCIAL ASPECTS OF ACUTE RHEUMATISM.

The social aspects of medicine have received much attention during the past few years, and as you will know the University of Oxford, helped by the generosity of the Nuffield Trustees, has recently founded a Chair of Social Medicine. Social medicine is no new subject. What is new is the increasing realization of its great importance to practitioners and students of all branches of medicine. It is difficult to give a definition of social medicine which is acceptable to everyone. Perhaps one of the best is, that it is that branch of medicine which concerns itself with the social environment and heredity of man as they affect health both mental and physical. Some people consider that social medicine is merely another name for public health work and preventive medicine. However, if we

B

accept the definition of social medicine, which I have suggested, then preventive medicine is the executive counterpart of social medicine and may be defined as the putting into effect of measures designed to raise the general level of health of the community and to prevent disease. Whatever definition of Social Medicine is accepted it is clear that its study covers a wide field. As Sir Farquhar Buzzard (1943) said recently, " it might be said to embrace the whole art and science of Medicine, the main object of which is to ensure that every member of the community may play his or her part in its life to full capacity and without the handicaps arising from mental or physical ill-health." Dr. Long Fox was a physician with views and ideas far ahead of his time in realizing that medicine must concern itself with social matters, and for this reason I hope that he would have approved the subject which I have chosen for this lecture. When President of the British Medical Association in 1894, he gave his presidential address on " the Medical Man and the State." In this address he said : " the poor, the sick and the criminal are our daily study primarily for the relief of the individual, but with nobler and further reaching aims—namely, that poverty may be mitigated by more healthy surroundings ; that sickness may be diminished by the education of the nation in the wiser laws of health." Perhaps I can best illustrate the different view points of curative and social medicine by an example recently used by Professor Henry Cohen (1943), in another connection. Suppose we are confronted by a young man of thirty who has developed paralysis of one side of the body. We examine him and decide that this paralysis is due to a clot in one of the arteries of the brain. However, in order to institute efficient treatment we must endeavour to discover the cause of the clot, and we find that this is due to disease of the artery caused by syphilis. At this point curative medicine is satisfied since the therapeutic indications are clear. Social medicine on the other hand asks why did this young man contract syphilis five years before. We do not know the complete answer to this question; it is obviously many-sided, and it does not require much imagination to envisage the story of poverty, poor housing, unattractive work and frustrated ambitions that may lie behind the final tragedy.

Acute rheumatism, the social aspects of which I wish to discuss to-night, is a disease which is very unfortunately named. Rheumatism suggests a condition of pain in or around a joint; yet acute rheumatism may run a fatal course with no joint pain of any sort. It is a disease which has different manifestations. First of all there is the arthritis or acute inflammation of joints from which the disease was named. Another manifestation is, strangely enough at first sight, Sydenham's chorea or St. Vitus's Dance. Why one patient should suffer from arthritis and another from St. Vitus's

Dance though both apparently are afflicted with the same disease is only one of the many baffling and intriguing mysteries of this condition. Dr. Long Fox by his researches into the nature of St. Vitus's Dance helped to lay one of the stepping stones whereby we finally learnt the association between this condition and acute rheumatism. In 1870, and again in 1884, he described fatal cases of chorea in which severe cardiac lesions were discovered after death. This brings us to another and the most important manifestation of acute rheumatism, that is an acute inflammation of the heart or carditis. This is much more serious than any other feature of the disease. The arthritis and St. Vitus's Dance both clear up completely and, while unpleasant in the acute phase, leave behind no permanent sequelæ. It is far otherwise with the carditis. Although recovery takes place, permanent scars are left in the heart which, sooner or later, lead to cardiac failure and finally the death of the patient.

Acute rheumatism is essentially a disease of childhood, and the vast majority of cases develop first during the years of school life from five to fifteen. Once a child has had an attack of acute rheumatism, instead of being immune to further attacks, as after an attack of diphtheria, he or she is very likely to develop a fresh attack within two or three years. It is these repeated attacks which leave the child at the age of sixteen or seventeen with a severely damaged heart. Not all cases of acute rheumatism show clinical evidence of involvement of the heart. However, there is reason to believe that the heart is affected in many cases where no abnormality is detected by ordinary examination. Some of you will remember that when Dr. Carey Coombs gave this lecture in 1925, he drew your attention to the main causes of heart disease and showed you the important part played by acute rheumatism. As a result of Dr. Carey Coombs's investigation in Bristol we were enabled by the Harmsworth Trustees to make a similar study in a number of large teaching hospitals throughout Great Britain. The figures collected then show that 38.4 per cent. of all cases of heart disease admitted to these hospitals in 1932 were due to acute rheumatism (Perry, 1934). The condition is, however, in many ways rather worse than this since the other main types of heart disease develop relatively late in life, whereas rheumatic heart disease incapacitates and kills at the prime. A study of the Registrar General's returns show that the vast majority of cases of death from heart disease under forty are due to acute rheumatism.

It has long been realized that there are striking differences in the incidence of acute rheumatism amongst different sections of the population. It became increasingly clear that the better-off members of the community were much less liable to this unhappy disease than the poor.

Sir Frederick Still, in 1927, found that of 1,000 consecutive out-patients seen at King's College Hospital Out-Patient Department 13.1 per cent. of those aged 6 to 10 showed evidence of rheumatic infection, whilst among 700 consecutive patients of the same age seen in private consulting practice only 5 or 0.7 per cent. showed signs of the disease. In 1930 Dr. Alison Glover (1930) stated that "no disease has a clearer cut social incidence than acute rheumatism, which falls perhaps thirty times as frequently upon the poorer children of the industrial town as upon the children of the well-to-do." This striking social incidence holds good all over the world, and in a study made in New South Wales in 1937 by Maddox (1937) it was found that in Sydney by far the majority of the patients came from the poorer industrial quarters of the city and very few from the better class suburbs. Apart from the social incidence it appeared that urban populations are more susceptible than rural. As a result of a discussion on the incidence of acute rheumatism at the Portsmouth meeting of the British Medical Association in 1923, a sub-committee was appointed to investigate the problem further. This sub-committee finally advised that an enquiry into the environment under which rheumatic heart disease arose in childhood should be conducted in an area defined as that included within the boundaries of the three counties of Gloucestershire, Somersetshire and Wiltshire. This enquiry was made during the three years from October, 1927, to September, 1930, and was aided by a grant from the Medical Research Council. It revealed a striking difference between Bristol and the surrounding counties. In Bristol there was found 7.72 cases for every 1,000 school children, whereas in Bath the incidence was 1.28, in Gloucestershire 1.03, in Somersetshire 2.17 and in Wiltshire 1.5 (Savage 1931). Thus we now had definite evidence that there was something in the social conditions prevailing in Bristol which was conducive to rheumatic heart disease. Since Bristol is a fairly large city and there are, within the city itself, very varying environmental conditions it appeared desirable to analyse the incidence in Bristol in more detail. This analysis was made on the basis of the City wards, since this was the only small unit for which information as to population was available (Perry and Roberts, 1937). It revealed the striking fact that in Redcliffe 3.87 cases occurred during the three-year period of the survey for every 1,000 persons and that in the same period the incidence in Redland was 0.15 cases per 1,000. Thus there was a larger difference between different areas of the city than between Bristol and the surrounding counties. We tried to find some other factor about which information was available in the different wards of the city which might show a similar relationship and we soon found that the degree of overcrowding expressed as persons per room showed a close correlation. In fact, Dr. Fraser Roberts who studied the figures stated that on the average the incidence of rheumatic heart disease per ward rises steadily in a linear manner as the density of persons per room increases.

This clear-cut relationship to overcrowding does not necessarily mean that the overcrowding in itself is the cause of the increased incidence of the disease. Over-crowding is merely one manifestation of poverty and the larger the family income the more rooms they are likely to inhabit.

Studies made before the Bristol investigation and published by the Medical Research Council (1927), while agreeing that there are marked social differences in the incidence of acute rheumatism, suggested that it is not the poorest members of the community who suffer most severely from the disease, but those slightly better off. It was said that the family in receipt of poor-law relief was less susceptible than the family just managing to make both ends meet. However, all these studies were open to serious criticism as to the suitability of the controls used. In most cases these were hospital out-patients not suffering from acute rheumatism, but it is clear that these patients were derived from much the same economic level. It therefore appeared desirable to check these findings and to obtain further and more accurate information. In 1937 the University of Bristol with the help of the Colston Research Society embarked on a social survey of the city. This provided a magnificent opportunity to investigate fully the social incidence of acute rheumatism. The social survey would provide us with the perfect control since we should know exactly the social status of a complete cross-section of the population of Bristol. Further, owing to the organization of the school medical department and the consultative rheumatic clinic at the General Hospital we had accurate information about nearly every rheumatic child in the city. The Medical Research Council came to our help and met the cost of extending the social survey to include all the rheumatic families. In order to avoid errors of diagnosis rheumatic heart disease, which can be fairly easily and definitely diagnosed, was chosen as the criterion of rheumatic infection. The survey included all families containing one or more children between the ages of 5 and 14, who had shown evidence of active rheumatic heart disease in the preceding eighteen months. You will remember that the general social survey only concerned itself with the families of manual workers and black coated workers with incomes not exceeding £5 per week (Tout, 1938). After the rheumatic families had been surveyed Mr. Tout rang me up and complained that I had removed from the list of cases which I had provided nearly all those above this income limit. Actually I had exercised no selection, but had included all cases as already defined. Three or four families only proved, when visited, to have incomes in excess of the limit. One or two families had left Bristol in the interval between the illness and the survey, but in all information was obtained from 341. From the sample of the total working-class population, families containing one or more children of school age were abstracted to serve as controls for the rheumatic families. The total number of such families was 1,424.

The final analysis of the information obtained in this way has been published by Dr. Daniel in the Journal of the Royal Statistical Society (1943). Dr. Daniel was able to investigate the relationship between the incidence of rheumatic heart disease and the net income of the family. By the net income is meant the money available for expenditure on food, clothes and fuel after that spent on fixed items, such as rents, rates, travelling expenses, insurance, trade union subscriptions and other regular payments has been subtracted. It is clearly not the total family income which is likely to affect susceptibility to disease as much as the income of the family relative to its needs. Thus the total income must be adjusted according to the size of the family and the age and sex of its members. In the social survey the minimum needs of each family was calculated, with slight modifications, according to the standards laid down by George. Dr. Daniel also studied the relationship with overcrowding and also the use of basement rooms, receipt of meals or milk at school and the membership of a doctor's club.

Taking the net income first. The families were divided into eight groups according to the net income expressed as a percentage of the minimum needs. There is a marked relationship between net income and incidence of the disease, those families with an income below the calculated minimum need showing 40 per cent. more rheumatic heart disease than the average working-class families and approximately twice as many as those in the highest income group. It may be urged that the numbers studied were so small that this might be a chance distribution. In order to eliminate as far as possible this risk the figures were studied statistically by subjecting them to the standard X2 test. This shows that the probability of this distribution being due to chance is less than one in a hundred.

Turning now to the question of overcrowding, the new survey showed that families with less than 0.6 rooms per person had about four times as many cases of rheumatic heart disease as those with 1.8 rooms or more. There is thus a clear inverse relationship between the incidence of the disease and rooms per person.

In the analysis of the figures for basement rooms, receipt of milk or meals at school, and the membership of a doctor's club the figures were so small that it was not possible to make any very definite comparisons. However, they suggest that membership of a doctors' club was associated with a low incidence, but receipt or non-receipt of meals or milk at school appeared to be associated with little difference in incidence ; while families using basement rooms had a slightly higher incidence, but one which is not statistically signficant. Dr. Daniel concluded that in Bristol in 1937 if the standards of the 30 per cent. of the working-class population with the most inadequate incomes and housing accommodation were raised to the average level of the rest of the working-class population, a decrease of 21 per cent. in the number of cases of rheumatic heart disease could be expected. And if standards of all were raised to the level of the highest 10 per cent. of all working-class families, the incidence of the disease would be roughly halved. One of the few good things which has emerged as a result of the present war is that the standards of living of the less well off has been raised and coincidentally with this there has been a marked reduction in the incidence of the disease. This wartime decline in acute rheumatism has recently been studied by Dr. Alison Glover (1943), who draws attention to the fact that there has been a steady decline since 1934, and that this decline has been accelerated during the war years. Thus in 1934 there were nearly 550 deaths from rheumatic fever, whereas in 1941 this figure had fallen to less than 200. These figures are derived from the Registrar General's returns for the whole country. A similar trend has been seen in Bristol where the incidence amongst school children was 3.9 per 1,000 in the two years 1938–39, and only 1.4 per 1,000 in 1941–42.

While the close association between the incidence of acute rheumatism and the standard of living of the community is clear the actual factor responsible for this association is unknown. In these days of the realization of the important part played in the maintenance of health by an adequate diet with a proper complement of vitamins and other essential food factors it is tempting to blame some dietetic deficiency. However, no definite evidence of this has been found despite several dietary surveys. There are some who believe that the overcrowding itself is at fault by facilitating frequent upper respiratory infection.

There is no doubt that infection plays a large part in the causation of acute rheumatism and I should be wrong if I gave you the impression that social conditions are the only important factor. While no one has succeeded in demonstrating any causal organism, as in the case of typhoid fever, we have learnt that in certain patients an upper respiratory infection, a simple sore throat or tonsillitis, due to a certain organism, the hæmolytic streptococcus, is often followed, after ten to thirty days interval of apparent good health, by an attack of acute rheumatism. The infection appears to pull the trigger when the gun has been suitably loaded.

Another factor of importance is heredity. Some families have a definite predisposition to the disease ; a very good example of this is the pedigree recently published by Dr. Pickles. Nevertheless it is interesting to note that when children from the social class in which acute rheumatism reaches its highest incidence are removed from their home environment the incidence is considerably reduced. Thus the Medical Research Council report showed that children living in certain poor-law residential schools were relatively free from rheumatic infection compared with children of their own class who were living at home. Newman found that amongst the mentally deficient children at Fountain Hospital, Tooting, the incidence of acute rheumatism was 0.4 per cent. whereas Glover estimated that amongst the elementary school children from which the inmates of Fountain Hospital were drawn it was at least 2 per cent. Professor Nixon drew attention in 1934 to the very low incidence of the disease at Stoke Park Colony where there are some 1,500 inmates. This apparent immunity to the disease of the children at Stoke Park has persisted and Professor Nixon tells me that he has never seen a case of a first attack of acute rheumatism in the Colony and that Dr. Bates, the Medical Superintendent, has no records of any such case. Such observations as these suggest that however large a part heredity may play in the production of the disease the environment of the child is far more important.

Of the various factors known to play a part in the causation of this serious disease the social environment is the only one capable of control. At the moment we have no means of preventing streptococcal infection in the general population, although there is some hope that we shall in the near future be able to control this infection in small groups. Heredity is clearly beyond any hope of control in the present state of our knowledge but standards of living can be improved.

Apart from the important rôle of environmental conditions in the causation of acute rheumatism social conditions create further difficulties for the patient. When children with rheumatic heart disease leave school many of them have great difficulty in obtaining suitable employment. Some of them are backward as a result of lost schooling through illness, but the more fortunate will have escaped this drawback if they have been treated at a properly equipped hospital school like Winford. There are, however, at the moment only a few such institutions. The majority of these children have hearts which, although damaged, are quite adequate for their immediate needs. They have no symptoms and are able to lead fairly normal active lives. It is clearly undesirable for them to enter laborious occupations, but they are well able to do ordinary factory and clerical work. However, there are two difficulties in their way under ordinary peacetime conditions. All grades of the civil service and many similar types of employment are closed to them by reason of the superannuation schemes. They fail to pass the ordinary medical examination and are rejected. Even where no such scheme exists the larger and better factories insist on a medical examination on entry, partly as a safeguard against the risks of employers' liability. Again these children are rejected although perfectly capable of doing good and useful work. As a result they drift into unsuitable occupations, the boys often becoming errand boys where they are exposed to all winds and weathers and have no future prospect to look forward to. Under wartime conditions these difficulties have largely disappeared since nearly anyone is able to obtain employment. But even now this is not always suitable. It should be realized that nearly everyone, however crippled, is capable of doing some work of value to the community. Under the necessity of compensating for war-time shortage of man power, the United States Civil Service Commission, the central recruiting agency for the Federal Government, has shown that thousands of jobs in industrial establishments of the government, such as arsenals, can be filled by judicious placing of physically handicapped persons. (Harvey & Luongo, 1943). An attempt has been made to work out a system of grading according to the disability suffered, including different types of heart disease. This, of course, necessitates an accurate medical assessment as a preliminary. It is to be hoped that that part of the Beveridge Report which recommends vocational training and subsequent employment for the physically handicapped will considerably lighten the lot of the unfortunate child suffering from rheumatic heart disease.

I must apologize if, in this short survey, I have paid undue attention to studies made in Bristol, but I felt that a Bristol audience would be interested to hear of this work. I have attempted to show you how social conditions help to cause an important disease and afterwards may make the life of a sufferer from that disease unnecessarily difficult. In this way I hope that I have carried out the exhortation of Dr. Long Fox when, in the address to which I have already referred, he told us that as medical men we should "concern ourselves with the well-being of the masses of mankind, the possibilities of the advancement of the race, the annihilation of all things that militate against the progress of the nation."

### REFERENCES.

Buzzard, F. (1943). *Practitioner*, cli. 129.

Cohen, H. (1943). *The Nature Method and Purpose of Diagnosis*, University Press, Cambridge.

Daniel, G. H. (1942). *Jour. Royal Statistical Soc.*, cv. 197.

Glover, A. (1930). *Lancet*, i. 499.

Glover, A. (1943). *Lancet*, ii. 51.

Harvey, V. K. and Luongo, E. P. (1943). *Jour. Am. Med. Assoc.*, cxxi, 100.

Long Fox, E. (1870). *Med. Times and Gaz.*, i. 601.

Long Fox, E. (1884). *Brit. Med. Chir. Jour.*, ii. 73.

Long Fox, E. (1894), *Brit. Med. Jour.*, ii. 237.

Maddox, K. (1937). *Med. Jour. Aust.*, 394.

Nixon, J. A. (1934). *Proc. Royal Soc. Med.*, xxvii., 969.

Perry, C. B. (1934). *Brit. Med. Jour.*, i. 278.

Perry, C. B. and Roberts, J. F. (1937). *Brit. Med. Jour.*, 2nd Supp., 154.

Pickles, W. N. (1943). *Lancet*, ii. 241.

Savage, W. G. (1931). *Brit. Med. Jour.*, 2nd Supp., 37.

Still, F. (1927). *M.R.C. Spec., Rep.*, No. 114. " Social Conditions and Acute Rheumatism."

Tout, H. (1938). *The Standard of Living in Bristol.* Arrowsmith, Bristol.

